# Pivotal role of intensivist despite low staffing

**DOI:** 10.1186/2197-425X-3-S1-A868

**Published:** 2015-10-01

**Authors:** AM Burki, L Noor, N Ali

**Affiliations:** Combined Military Hospital Peshawar, Anaesthesia and Intensive Care, Peshawar, Pakistan; Combined Military Hospital Peshawar, Obs & Gynae, Peshawar, Pakistan; Combined Military Hospital Peshawar, Pathology, Peshawar, Pakistan

Appointment of an intensivist to the nursing staff for better patient outcome with low level of nursing care has not been challenged before in studies. An intensivist not only gives plan for the patient management but also can educate the nursing staff for better patient outcome.

## Objectives

The purpose of this study is to establish the effect of appointing an intensivist to low nursing staff on ICU mortality.

## Methods

This retrospective observational study is conducted in intensive care unit of combined military hospital Peshawar. Total of 2041 patients are included in this study during a period from 2011 to 2014. Only those patients were included in the study who were 12 years or older, those who require organ support and had ICU stay for at least 24 hours . Patients with advanced disease who require palliative care only, those who require CABG were excluded from the study.

Data is collected from admission and discharge book maintained at ICU. Patients are divided in two groups: group A includes patients who received nursing care without an intensivist and group B includes patients who received nursing care under supervision of an intensivist.

## Results

Of total 2041 patients 54% were men.The mean age was 58 years. Each group has patients with medical and surgical ailments.Outcome is measured interms of mortality. Mortality in group A is 23.01 to 33.02 % and in group B is 20.29 to 23.60 % that is statistically significant and support our hypothesis that appointment of intensivist to nursing staff is associated with low mortality and better patients outcome,despite of low human resource.

## Conclusions

In our retrospective cross sectional study addition of an intensivist to the nursing staff has improved the mortality rate to about 20.29 to 23.60 % which is comparable to the international values 11 to 18%.

## Grant Acknowledgment

Professor Nadir Ali

Dr Lubna Noor
